# Bacteriological etiology and treatment of mastitis in Finnish dairy herds

**DOI:** 10.1186/s13028-017-0301-4

**Published:** 2017-05-25

**Authors:** Johanna Vakkamäki, Suvi Taponen, Anna-Maija Heikkilä, Satu Pyörälä

**Affiliations:** 1 Department of Production Animal Medicine, Faculty of Veterinary Medicine, University of Helsinki, Paroninkuja 20, 04920 Saarentaus, Finland; 2 Natural Resources Institute Finland (Luke), Koetilantie 5, 00790 Helsinki, Finland

**Keywords:** Bovine mastitis, Bacteriology, PCR, Treatment, Culling

## Abstract

**Background:**

The Finnish dairy herd recording system maintains production and health records of cows and herds. Veterinarians and farmers register veterinary treatments in the system. Milk samples for microbiological analysis are routinely taken from mastitic cows. The laboratory of the largest dairy company in Finland, Valio Ltd., analyzes most samples using real-time PCR. This study addressed pathogen-specific microbiological data and treatment and culling records, in combination with cow and herd characteristics, from the Finnish dairy herd recording system during 2010–2012.

**Results:**

The data derived from 240,067 quarter milk samples from 93,529 dairy cows with mastitis; 238,235 cows from the same herds served as the control group. No target pathogen DNA was detected in 12% of the samples. In 49% of the positive samples, only one target species and in 19%, two species with one dominant species were present. The most common species in the samples with a single species only were coagulase-negative staphylococci (CNS) (43%), followed by *Staphylococcus aureus* (21%), *Streptococcus uberis* (9%), *Streptococcus dysgalactiae* (8%), *Corynebacterium bovis* (7%), and *Escherichia coli* (5%). On average, 36% of the study cows and 6% of the control cows had recorded mastitis treatments during lactation. The corresponding proportions were 16 and 6% at drying-off. For more than 75% of the treatments during lactation, diagnosis was acute clinical mastitis. In the milk samples from cows with a recorded mastitis treatment during lactation, CNS and *S. aureus* were most common, followed by streptococci. Altogether, 48% of the cows were culled during the study. Mastitis was reported as the most common reason to cull; 49% of study cows and 18% of control cows were culled because of mastitis. Culling was most likely if *S. aureus* was detected in the milk sample submitted during the culling year.

**Conclusions:**

The PCR test has proven to be an applicable method also for large-scale use in bacterial diagnostics. In the present study, microbiological diagnosis was unequivocal in the great majority of samples where a single species or two species with one dominating were detected. Coagulase-negative staphylococci and *S. aureus* were the most common species. *S. aureus* was also the most common pathogen among the culled cows, which emphasizes the importance of preventive measures.

## Background

During recent decades, considerable structural changes have occurred in the dairy industry in many countries. In Finland, the total number of dairy farms has decreased substantially but average herd size and numbers of farms with automatic milking systems (AMS) have increased [1–3]. With increasing herd size, good management practices and care of the cows become ever more important [4, 5]. Despite decades of mastitis control programs in countries with an intensive dairy industry, mastitis continues to be the most common and economically important disease of dairy cows. It considerably affects milk production, animal welfare, and food safety [6–8]. Today’s consumers make increasing demands for adequate animal welfare as well as food quality and safety, which also must be taken into account on modern dairy farms [9].

Mastitis results from intramammary infection (IMI), mostly caused by various bacterial species. Knowledge on the bacteriological etiology of IMI is important for efficient mastitis control and treatment [10, 11]. Distribution of the pathogens causing IMI varies among countries and herds. The most common causes of IMI are the major pathogens *Escherichia coli*, *Staphylococcus aureus*, and environmental streptococci, and the minor pathogens coagulase-negative staphylococci (CNS) [12–15]. Previous studies on the microbiological etiology of mastitis or IMI were mainly based on sampling selected herds in different countries. In Finland, national surveys with a random sample from all Finnish dairy herds were published [16]. In a previous Finnish study, Koivula et al. [17] used a smaller, culture-based bacteriological database of similar type as in this study. Some studies investigated the relationship between herd factors, such as general management, milking technique or production type, and distributions of mastitis-causing bacterial species in the herds [15, 18–20]. To our knowledge, studies combining pathogen-specific bacteriological mastitis data, treatment and culling records, and cow and herd characteristics from large national databases have not been carried out.

The Finnish dairy herd recording system has long traditions: it began in 1898 [21]. In 2015, the recording system covered 74% of dairy herds and 81% of dairy cows [22]. In herds belonging to this system, production records, including milk yield, milk protein content, milk fat content and milk somatic cell count (SCC), are recorded for every cow. The national health monitoring of dairy herds, constituted during the early 1980s, keeps health records for every Finnish cow. Veterinarians and farmers register all veterinary treatments per cow or herd in this system. The Nordic dairy herd health recording system is globally unique. In many countries, mastitis treatments are not recorded or are recorded at herd level only, or in connection with a specific health project. Unfortunately, the completeness of the records for clinical mastitis in Finland, according to a study carried out in 2008, was only 56%, which was lower than in the other Nordic countries [23].

In Finland, milk samples for bacteriology are routinely taken in most cases of mastitis [24]. The laboratory of the largest dairy company in Finland, Valio Ltd., analyzes the majority of these samples: 78% of Finnish dairy producers send milk samples to this laboratory (Personal communication Kristiina Sarjokari, Valio Ltd., 2016). Since 2010, a multiplex real-time polymerase chain reaction (PCR) assay has been used for the microbiological analyses.

Our first aim was to use a large microbiological database of quarter milk samples from mastitic dairy cows to study the bacteriological etiology of mastitis in Finland. Our second aim was to study treatment and culling records for the cows in the study herds collected from the databases of the Finnish dairy herd recording system. Detailed analysis of the relationships between pathogen-specific IMI and different cow- and herd-specific factors are presented in Taponen et al. [25]. The present study provides information about a PCR assay used for routine microbiological diagnostics of mastitic milk samples.

## Methods

### Microbiological data

Microbiological diagnoses of quarter milk samples from mastitic cows analyzed at the laboratory of Valio Ltd. during 2010, 2011 and 2012 were received from the database of Valio Ltd. For microbiological analyses, PathoProof™ Mastitis PCR Complete-12 assay (Thermo Fisher Scientific, Waltham, MA, USA) was used, which contained oligonucleotides for the staphylococcal β-lactamase gene (*blaZ*) and for the following microbial species or groups of species: *Corynebacterium bovis*, *Enterococcus* spp., including *Enterococcus faecalis* and *Enterococcus faecium*, *E*. *coli*, *Klebsiella oxytoca* and *Klebsiella pneumoniae*, *Serratia marcescens*, *Staphylococcus* spp., including all relevant CNS, *S. aureus*, *Streptococcus agalactiae*, *Streptococcus dysgalactiae*, *Streptococcus uberis*, and *Trueperella pyogenes* and *Peptoniphilus indolicus*. In March 2012, the test kit was changed to PathoProof™ Mastitis PCR Complete-16 assay, which also includes *Mycoplasma* (*M*.) spp., *Mycobacterium bovis*, *Prototheca* spp., and yeasts.

The reason for milk sampling was evidence of clinical or subclinical mastitis (elevated milk SCC) in the quarter. Milk sample was taken by herd staff or a visiting veterinarian using an aseptic technique [26]. In this study, a cow with a quarter milk sample with DNA of at least one target pathogen species in the PCR assay was considered to have IMI. These cows comprise the study group. Only samples with microbial DNA of one microbial species alone were included in calculation of the proportions of different pathogens. The control group comprised all other cows from the same herds: those for which no milk samples were sent for microbiological analysis to the laboratory of Valio Ltd.

Bacteriological data for 2010–2012 consisted of microbiological results from 240,067 quarter milk samples (Table 1). Samples were taken from 93,529 dairy cows in 4725 dairy herds. The control group consisted of 238,235 cows from the same dairy herds. In the analyses carried out on an annual basis, the same cow may appear several times.

**Table 1:** Number of quarter milk samples, cows sampled (study group) and control cows (no milk samples submitted) in 2010, 2011 and 2012

	2010	2011	2012	Total number of samples
Samples	64,630	84,413	91,024	240,067
Samples with one target pathogen	31,731	44,037	41,743	117,511

				Total number of cows	Total number of individual cows
Cows in the study group	32,723	41,949	45,582	120,254	93,529
Cows in the control group	177,409	177,205	180,427	535,041	238,235
Total number of cows	210,132	219,154	226,009	655,295	331,764

### Milk and health recording data

Milk and health recording data of the cows were received from the databases of the Finnish dairy herd recording system, and the Finnish cattle health monitoring system.

The data include information on individual cows for breed, age, parity, annual milk, fat and protein yield, results of test milking six to twenty-four times a year, the number of feeding days, date of diagnosis and treatment for mastitis (clinical, subclinical) or dry cow therapy (DCT), and date and reason for culling. The data also provide herd-specific information, such as that for milking and housing system and production type (organic vs. conventional) for 93% of the cows in the dataset.

In Finland and other Nordic countries, only veterinarians are permitted to prescribe antimicrobials for use in animals. According to Finnish legislation, veterinarians record treatment of a cow after administering products with a withdrawal period, such as antimicrobials. Treatment records are regularly submitted to the central cattle health database. In subclinical or mild clinical mastitis, herd staff often take a milk sample and send it for microbiological analysis and contact their veterinarian after receiving the microbiological result. Mastitis treatment in such cases may be prescribed without the veterinarian visiting the farm, and the herd staff should record the treatment for that cow. Veterinary-supervised cases are recorded using four different codes for mastitis during lactation: acute clinical, subclinical, chronic, and unspecified mastitis, and one code for DCT [27]. Herd staff use three additional codes to record treatment of mastitis: mastitis during lactation, DCT for mastitis, and DCT for prevention. Finnish legislation concerning antimicrobial treatments of production animals changed after the onset of this study. Dairy farmers now have limited access to antimicrobials to treat some predefined diseases like subclinical mastitis, on certain conditions. These herds have to belong to herd health recording system and have regular visits of a supervising veterinarian. Microbiological results of milk samples shall be available before treatment of mastitis.

The data were organized and analyzed using SAS 9.4 (SAS Institute Inc., Cary, NC, USA) and SPSS 22 (IBM SPSS Statistics, Armonk, NY, USA) software. Differences in the treatment records for mastitis between the study group and the control group were tested using a Chi square test.

## Results

### Characteristics of cows and herds

In our data, 61.9% of cows were Nordic Red (NR), also known as Finnish Ayrshire, 36.6% Holstein (HOL), and 1.8% Finncattle, Jersey, or different crossbreds. The mean parity was 2.4 in NR and 2.2 in HOL cows (range 1–15). The proportions of parities 1, 2, 3 and ≥4 were 33.8, 26.5, 18.4, and 21.3%, respectively. The average annual milk production of the cows was 8598 kg per cow, 8323 kg for NR and 9154 kg for HOL cows.

Most herds were in conventional production systems and only 1.9% of the cows were on organic farms. 1.6% of both NR and HOL cows were in organic production, but for Finncattle the proportion was 7.7%. The average herd size was 44.7, 46.5, and 48.0 in 2010, 2011, and 2012, respectively. The minimum number of cows in a herd was three for all years, but the maximum number increased from 444 in 2010 to 498 in 2012. The proportions of free-stalls and AMS increased during the study period. In the entire period, 51.8% of the cows were housed in tie-stall barns, 46.6% in different types of free-stall barns, and 1.6% in barns with different mixed systems. In total, 52.0% of the cows were milked with a pipeline milking system in tie stalls, 30.4% in a milking parlor, 17.0% with AMS, and 0.6% with a bucket milking machine in tie stalls.

### Microbiological results of the milk samples

DNA of some target pathogen species or group of species was detected in 87.6% of the quarter milk samples. 12.4% of the samples were negative, i.e. did not contain DNA of any target species. In 49% of the samples, DNA of only one target species or group of species, and in 25.2% two species or group of species, were detected. In samples with DNA of two bacterial species, a dominant species (>90% of the total target DNA in the sample) was present in 66.2% of the samples. In 13.4% of all samples, DNA of more than two bacterial species was found. In these samples, the number of target species or group of species detected in the sample was as follows: 3 in 9.2%, 4 in 2.9%, 5 in 0.9%, 6 in 0.3%, 7 in 0.07%, and 8 or 9 in less than 0.02%. One milk sample contained DNA from 10 target species or group of species.

The most common bacteria detected in milk samples were CNS and *S. aureus*. Results for milk samples with DNA of only one microbial species or group of species are shown in Table 2. Mean cycle threshold (Ct) values, reflecting the amount of pathogen DNA in the sample, ranged between 21.9 and 33.1 (Table 2). The staphylococcal *Bla*Z gene was found in 27.5% of samples with DNA of CNS and in 26.9% of samples with DNA of *S. aureus*.

**Table 2:** Number and proportion of samples with DNA of a target bacterial species or group of species detected alone in 117,511 quarter milk samples analyzed in the laboratory of Valio Ltd. in Finland during 2010–2012 using PathoProofTM Mastitis PCR Assay, and the mean cycle threshold (Ct) values

Pathogen	N	%	Mean Ct value^a^
CNS	50,836	43.3	31.5
*Staphylococcus aureus*	24,754	21.1	28.0
*Streptococcus uberis*	10,572	9.0	26.6
*Streptococcus dysgalactiae*	9292	7.9	27.0
*Corynebacterium bovis*	8510	7.2	31.9
*Escherichia coli*	5562	4.7	29.3
*Trueperella pyogenes/Peptoniphilus indolicus*	2921	2.5	26.0
Yeasts	1812	1.5	31.3
*Enterococcus* spp.	1722	1.5	30.7
*Klebsiella* spp.	836	0.7	28.9
*Streptococcus agalactiae*	501	0.4	25.6
*Serratia marcescens*	149	0.1	33.1
*Prototheca* sp.	34	0	32.7
*Mycoplasma* spp.	7	0	21.9
*Mycoplasma bovis*	3	0	27.1
Total	117,511	100	

^a^Ct value reflects the amount of pathogen DNA in the sample: the lower Ct value, the more DNA

In milk samples with two bacterial species or groups of species detected, with one dominant species, the most common dominant species were CNS (40.2%), *Str. dysgalactiae* (16.7%), *S. aureus* (14.4%), *Streptococcus uberis* (10.0%) and *T. pyogenes/P. indolicus* (5.3%). The most common species present in the sample together with the previously listed dominant species were *C. bovis* (29.6%), CNS (18.4%), yeasts (11.6%), *T. pyogenes/P. indolicus* (10.9%) and *S. aureus* (7.7%).

### Mastitis treatments

In total, 53,993 cows in the study group and 63,843 cows in the control group received treatment for mastitis, received DCT, or received both. The proportion of cows in the study and control groups treated for clinical or subclinical mastitis, or with DCT, and the number of treatments per cow are presented in Table 3. The proportions of cows treated during lactation and with DCT were significantly higher in the study group than in the control group (p < 0.001). Quarter milk samples for bacteriology were submitted to the laboratory on average from 56.4% of the cows with a recorded treatment during lactation during the same year; this percentage increased during the study period from 47.4 to 64.4%.

**Table 3:** Proportion of cows in the study group and control group treated during lactation or at drying-off (dry cow therapy) or both. Number of treatments per cow during the study year is also shown

Year	Cows treated during lactation		Cows treated at drying-off	Cows treated both during lactation and at drying-off	
	Cows, %	Treatments per cow	Cows, %	Cows, %	Treatments per cow
Study group					
2010	31.0^a^	1.31	9.4^c^	6.8^e^	2.48
2011	29.4^a^	1.28	9.6^c^	6.6^e^	2.45
2012	27.7^a^	1.27	9.2^c^	5.8^e^	2.40
Average	29.3	1.29	9.4	6.4	2.44
Total number of treated cows	35,074		11,287	7632	
Control group					
2010	6.7^b^	1.23	5.2^d^	1.0^f^	2.35
2011	5.4^b^	1.21	5.1^d^	0.7^f^	2.32
2012	4.1^b^	1.19	4.6^d^	0.6^f^	2.31
Average	5.4	1.21	5.0	0.8	2.33
Total number of treated cows	28,976		30,762	4105	

Significance of difference: a, b, c, d, e, f, p < 0.001

Proportions of different mastitis diagnoses of the study group cows among the veterinary-supervised mastitis treatments reported in the health recording system in 2012 were as follows: acute clinical mastitis 75.2%, subclinical mastitis 9.6%, chronic mastitis 2.0%, unspecified mastitis 7.4%, and mastitis noted by herd staff 5.7% (mastitis during lactation). The respective figures in the control group were 77.7, 8.7, 2.1, 7.4 and 4.1%. Cows in the study group and treated after diagnosis of acute clinical mastitis, had most often the following species or group of species in their milk sample during the treatment year: CNS (22.6% of the cows), *S. aureus* (22.5%), *Str. uberis* (9.99%), *Str. dysgalactiae* (9.02%), *E. coli* (5.5%), and *C. bovis* (3.6%). For cows in the study group and treated for subclinical mastitis during the year, the order of the species was the same, with the exception of *E. coli* and *C. bovis:* The proportion of cows having *C. bovis* was higher than that of *E. coli*. The share of cows having CNS was 31.5%.

### Culling of cows

Altogether 48.1% of the individual cows were culled during the 3-year study period. The reason for culling was reported for 88.5% of those cows. Among the reported reasons, the main causes were mastitis (23.6%) and poor fertility (19.0%). In the study group, mastitis was reported as the reason for culling for 49.1% of the culled cows for which the culling reason was reported. In the control group, the respective proportion was 18.2%. Table 4 shows the numbers and proportions of cows culled due to mastitis. The pathogens detected most often in the milk samples of the culled cows are also presented in the table.

**Table 4:** Number and proportion of the cows culled with mastitis as the reported culling reason in the study group and the control group, and the most common pathogens detected alone in the milk samples of the cows in the study group during the same year

	2010		2011		2012	
	Number of culled cows	%	Number of culled cows	%	Number of culled cows	%
Study group	3048	50.1^a^	4461	49.6^a^	4599	47.9^a^
CNS	581	19.1^b^	949	21.3^b^	866	18.8^b^
*Staphylococcus aureus*	888	29.1^b^	1383	31.0^b^	1239	26.9^b^
*Streptococcus uberis*	210	6.9^b^	336	7.5^b^	294	6.4^b^
*Streptococcus dysgalactiae*	168	5.5^b^	303	6.8^b^	221	4.8^b^
*Corynebacterium bovis*	143	4.7^b^	225	5.0^b^	197	4.3^b^
*Escherichia coli*	148	4.9^b^	238	5.3^b^	222	4.8^b^
Control group	7825	19.9^a^	6953	18.0^a^	6455	16.8^a^

^a^Proportion of cows from the total number of cows culled and culling reason reported in study group and in control group, respectively

^b^Proportion of cows having the respective pathogen in their milk sample from the total number of culled cows in study group

## Discussion

This study reports bacteriological results from bovine intramammary infections using information from more than 240,000 quarter milk samples analyzed with real-time PCR. Finland is to our knowledge the only country where PCR testing has largely replaced conventional culturing in routine microbiological diagnostics of quarter milk samples from mastitic cows [28, 29]. The PCR method used here identifies a wider range of target pathogens than commonly revealed by routine conventional culturing [24]. The uniform diagnostic method used and the large numbers of samples allowed us to make confident inferences on the microbiological etiology of mastitis in Finland.

The majority of the milk samples contained DNA of some target species or group of species. The proportion of negative target DNA samples, 12.4%, was lower than the proportion of “no growth” samples in studies using conventional bacteriology, which generally has ranged from 20 to 40% [13, 15, 17, 29]. This is at least partly explained by the capacity of the PCR method to detect also DNA of growth-inhibited or dead microbes [30]. The samples with DNA of some target species were largely easy to interpret because in 49.0% of the samples DNA of only one target species or group of species was detected. This agrees with previous reports where the same PCR test was used with smaller materials [24, 28]. In samples with DNA of two bacterial species (25.2%), a dominant species, being the likely causal agent of IMI, was found in 66.2% of the samples. Thus, in 75% of all samples, the causal pathogen could be identified and the result was unequivocal. These results can be considered diagnostically valid [24]. In 13.4% of the samples, DNA of more than two bacterial species was detected, up to ten species in one sample. Currently, reporting of PCR results differs from that for conventional culturing in that all target species detected in the sample in DNA quantities above the cycle threshold cut-off for the test are reported whereas in culturing, samples with three or more species growing on the plate are discarded, the plate being considered contaminated [26]. Sporadic colonies of minor pathogens or common contaminants on a plate with growth of a major mastitis pathogen are commonly ignored [24]. The same interpretation system could be applied also in PCR diagnostics [31]. In general, PCR testing has proven to be a fast, sensitive and reliable test suitable for routine mastitis bacteriology [24, 28, 32]. An advantage of the PCR test is the comprehensive panel of pathogens, and uniform procedure and interpretation, in contrast to bacteriological culturing, where performance and procedures of laboratories may differ [33]. High costs of molecular methods such as PCR may still limit their use for mastitis diagnostics in many countries.

Our pathogen distributions (Table 2) can be compared with those from previous studies even though others used conventional culture methods. In the present study, the most common bacterial species in the samples with DNA of only one target species or group of species were CNS (43.3%), followed by *S. aureus* (21.1%). The proportion of CNS agrees with previous findings in the Finnish national survey, where 49.6% of the isolates were CNS [16], but is much higher than in another Finnish study by Koivula et al. [17], who reported that CNS caused 18% of clinical and 24% of subclinical mastitis. In the study by Pitkälä et al. [16], the samples were taken from all quarters of all cows in the herds and thus the findings for bacterial infection were mainly associated with subclinical mastitis or teat end colonization. Our high proportion of CNS may partly be explained by the sensitivity of the PCR assay as compared with conventional culturing [24]. The high average Ct value for samples with CNS, generally indicating little DNA in the samples, also supports this view (Table 2).

The most common bacteria found in the milk samples of cows with recorded treatment of clinical mastitis were CNS and *S aureus,* with equal shares. The amount of CNS detections in milk samples (in total) was double compared to that of *S. aureus* detections (Table 2), showing that most *S. aureus* detections but only part of CNS detections lead to treatment of clinical mastitis. CNS are considered to be minor pathogens but are able to cause clinical mastitis, mostly mild [34]. It is possible that with changes in the dairy industry CNS currently cause more clinical mastitis than before, but another more likely explanation may be that herd staff are sensitive to react to mastitis with mild or transient clinical signs, aiming to keep bulk milk SCC low. Clinical mastitis is a broad term that includes all cases with any visible clinical sign, including mild changes in the milk appearance or consistency [35]. The high proportion of CNS in our study was not surprising because they have become very common IMI- and mastitis-causing agents in Finland and many other countries [15, 36, 37].

In our study, the proportion of *S. aureus* (21%) was higher than in the Finnish national survey (10%) [16]. It was similar to the proportion reported for clinical mastitis in Sweden [38], but high compared with studies from other countries [12, 15, 39, 40]. The high proportion of *S. aureus* recorded here means that this pathogen continues to be extensively responsible for mastitis problems in Finnish dairy herds. The proportions of *Str. uberis* (9.0%), *Str. dysgalactiae* (7.9%) and *E. coli* (4.7%) were similar to the results of Koivula et al. [17]. It is difficult to compare our proportion of *C. bovis* (7.2%) with that for other studies, in particular with those on clinical mastitis, because *C. bovis* is seldom reported. In the national survey, its proportion was over 34%, but then all cows and quarters in the study herds were sampled [16]. In samples with two bacterial species with one dominant species the most common concomitant species detected were *C. bovis*, CNS, yeasts, and *T. pyogenes/P. indolicus*. All these species are potential contaminants that originate from the teat end or barn environment, and their presence in milk samples is mostly of no clinical importance.

A clear difference between our results and those from other studies is the much higher proportion of environmental pathogens in many other countries. In studies from the UK, *Str. uberis* were the species most commonly isolated, followed by *Enterobacteriacae* [12, 14]. In another countries, environmental streptococci were the most common finding in clinical mastitis [13, 39]. *Str. agalactiae* was rarely detected in this study (382 cows). These samples originated mainly from a few large herds: 57% of the samples were from herds with more than 99 cows and 27% from herds of 69–99 cows. Many samples came from the same cows: 22% of *Str. agalactiae*-positive cows had at least two positive samples (one to five positive samples) (data not shown). The herds with sampled cows were probably included in eradication programs. We conclude that proportions of mastitis-causing bacterial species always differ among countries and herds due to different environmental and management factors [13, 18, 41].

The Finnish cattle health monitoring system is mainly based on records of diagnoses and treatments made by veterinarians [42]. In contrast to the situation in many other countries, in Finland, as in all Nordic countries, antimicrobials are prescription-only medicines and strictly controlled by veterinarians. The current Finnish legislation on medical treatment of animals emphasizes the importance of microbiological diagnosis before any antimicrobial treatment of dairy cattle. In most cases when herd staff identifies a cow with clinical mastitis they contact their veterinarian who visits the farm. In cases of subclinical and mild clinical mastitis, herd staff first take a milk sample and then contact their veterinarian. Such cases do not necessarily receive antimicrobial treatment. In our study, on average 35.7% of the cows in the study group and 6.2% of the control cows had a recorded mastitis treatment during lactation. The significant difference between the sampled study cows and the control cows was expected.

In Finland, based on the numbers of milk samples, total number of dairy cows and antimicrobial consumption figures, it can be estimated that a milk sample for bacteriology is taken in a majority of mastitis cases [43]. We found that quarter milk samples were submitted to the laboratory on average for nearly 60% and, in the last study year, over 60% of the cows with a recorded treatment during lactation. It must be pointed out that the recording completeness for veterinary-supervised cases of mastitis in Finland has not been 100%, but according to a Nordic study only 56% (95% confidence interval 48–64%) [23]. Consequently, treatment records may be missing from our data, although disease reporting for dairy cows probably has become more complete since 2008 when the study of Wolff et al. [23] was carried out. Disease recordings are now done through the national herd health system Naseva [44] with which control of treatments has improved.

Control cows with a recorded treatment were either treated without taking a milk sample or the sample was taken but not analyzed in the laboratory of Valio Ltd. In Finland, veterinarians sometimes use triplate agars for rapid diagnosis of acute clinical mastitis and the results are generally not recorded [45]. It is still likely that mastitis cases diagnosed and sampled by farmers and treated via a veterinary prescription may remain unrecorded; this is supported by the low figure for treatments recorded with the specific code for notes of herd staff for treatment (data not shown). Many cows are repeatedly treated. The average number of treatments per cow was 1.3 and treatments may further accumulate for some of the cows.

The number of cows with recorded treatments for subclinical or chronic mastitis during lactation was low, which complies with the Finnish guidelines for mastitis treatment, where it is generally advised to postpone treatment of subclinical mastitis during lactation until drying-off, and treatment of chronic mastitis is not recommended [46]. Another possibility is that these treatments, often given by herd staff after veterinary prescription, are not reported. DCT was administered on average to 9.4% of the study cows and to 5.0% of control cows, and respectively 6.4 and 0.8% of the cows had been treated both during lactation and with DCT. Based on the consumption figures for intramammary products in Finland, approximately 22% of cows receive DCT [43]. Thus a considerable part of the treatment records for DCT are missing from our data. Figures for proportions of cows treated both during lactation and at drying-off were unexpectedly low. In general, DCT is recommended for cows that have had mastitis during lactation [46], but seemingly this advice is not followed on Finnish dairy farms.

Identification of the causal agent of IMI is important for mastitis management and treatment, but the routine is still rare in many countries and herds. For instance, in the Netherlands, only 34% of farmers submit milk samples for microbial analysis from cows with clinical mastitis and 22% from those with subclinical mastitis [10]. Prevention strategies in the herds should be pathogen-specific because problems caused by different bacteria need different approaches [11]. Treatment of mastitis should be based on microbiological diagnosis to avoid inefficient or unnecessary use of antimicrobials [14, 47, 48]. Even simple on-farm diagnostics, which only classifies mastitis-causing agents into Gram-negative and Gram-positive, has proven to be useful and reduced treatment costs [49, 50].

In the study group, mastitis was the reason for nearly half of the culled cows; in the control group the respective proportion was lower, as expected. Culling because of mastitis generally represents premature culling, which constitutes one-fifth of the costs of clinical mastitis in Finland [8]. The comparison of the shares of the pathogens in Tables 2 and 4 indicates that *S. aureus* was more common among culled cows than in the whole sample data. Thus, presence of *S. aureus* means increased risk of culling the cow. This may reflect the often poor outcome for treatment of mastitis caused by *S. aureus* [51]. The shares of the rest five pathogens presented in Table 4 were similar or lower among culled cows than in the whole dataset indicating that only *S. aureus* is a real risk factor for culling due to mastitis. However, more detailed studies on pathogen-specific diagnoses and treatment data using our large database will be carried out.

## Conclusions

In Finland, quarter milk samples are routinely taken from cows with mastitis. Most are analyzed using a PCR-test which has proven to be an applicable method also for large-scale use in bacterial diagnostics. In the present study, microbiological diagnosis was unequivocal in the great majority of samples in which only one species or two species with one dominating were detected. The reporting of results with more than two species detected should be improved to make the interpretation easier. CNS and *S. aureus* were the most common species detected in the milk samples whereas the proportion of environmental pathogens was lower than in many other countries. *S. aureus* was also the most common pathogen among the culled cows, which emphasizes the importance of preventive measures. Mastitis treatments are probably often based on bacterial diagnosis, but the activity of dairy farmers to submit milk samples for bacterial analysis could be further increased. With pathogen-specific treatments, inefficient and unnecessary use of antimicrobials can be decreased, which also improves food safety.
